# Bias Stress and Temperature Impact on InGaZnO TFTs and Circuits

**DOI:** 10.3390/ma10060680

**Published:** 2017-06-21

**Authors:** Jorge Martins, Pydi Bahubalindruni, Ana Rovisco, Asal Kiazadeh, Rodrigo Martins, Elvira Fortunato, Pedro Barquinha

**Affiliations:** 1 CENIMAT/I3N, Departamento de Ciência dos Materiais, Faculdade de Ciências e Tecnologia (FCT), Universidade NOVA de Lisboa (UNL) and CEMOP/UNINOVA, 2829-516 Caparica, Portugal; jds.martins@campus.fct.unl.pt (J.M.); a.rovisco@campus.fct.unl.pt (A.R.); a.kiazadeh@fct.unl.pt (A.K.); rm@uninova.pt (R.M.); emf@fct.unl.pt (E.F.); 2 IIIT Delhi, Okhla Industrial Estate, Phase III, New Delhi 110020, India

**Keywords:** bias stress, a-IGZO TFTs, robust oxide TFT circuits, large-area flexible electronics

## Abstract

This paper focuses on the analysis of InGaZnO thin-film transistors (TFTs) and circuits under the influence of different temperatures and bias stress, shedding light into their robustness when used in real-world applications. For temperature-dependent measurements, a temperature range of 15 to 85 °C was considered. In case of bias stress, both gate and drain bias were applied for 60 min. Though isolated transistors show a variation of drain current as high as 56% and 172% during bias voltage and temperature stress, the employed circuits were able to counteract it. Inverters and two-TFT current mirrors following simple circuit topologies showed a gain variation below 8%, while the improved robustness of a cascode current mirror design is proven by showing a gain variation less than 5%. The demonstration that the proper selection of TFT materials and circuit topologies results in robust operation of oxide electronics under different stress conditions and over a reasonable range of temperatures proves that the technology is suitable for applications such as smart food packaging and wearables.

## 1. Introduction

InGaZnO thin-film transistors (IGZO TFTs) enable uniformity over large-areas, compatibility with low-cost and low-temperature fabrication techniques, and high mobility (>10 cm^2^/V·s), setting oxide TFTs as a winning alternative for flexible large area electronics (LAE) when compared to competing TFT technologies (e.g., a-Si:H and poly-Si) [1]. As a good maturity level of this transistor technology starts to be achieved, circuit design and fabrication [2,3,4,5] becomes increasingly important, enabling one to implement smart integrated systems—namely, near-field communication (NFC) smart labels [6], intelligent packaging, and systems on glass, plastic, or garments [7]. However, the continuous real-time operation of these applications demands robust circuit performance against bias stress and over a reasonable range of temperatures. Though IGZO TFTs show better stability compared to a-Si:H TFTs, several studies have demonstrated that they still show non-negligible threshold voltage ($V_{TH}$) shift with respect to bias stress [8,9,10] and temperature [11,12].

In oxide TFTs, $V_{TH}$ variation with gate bias stress is due to the charge trapping at the semiconductor dielectric interface or charge getting into the dielectric [8,9,10]. Due to the variation in total charge of the conductive channel, the drain current ($I_{DS}$) of the TFT will also be changed. On the other hand, as temperature increases, mobility increases and $V_{TH}$ tends to have a negative shift, implying that the semiconductor and hence the device are becoming more conductive. Possible causes are oxygen vacancies in the semiconductor creating additional states near conduction band and excitation of electrons due to temperature. IGZO TFTs have been analyzed under different temperature ranges to gain insights into the mechanisms controlling carrier transport. Chowdhury et al. worked in a low-temperature range (10–300 K), concluding that variable range hopping was dominant below 80 K, and that above this temperature thermally-activated band conduction would dominate [12]. Between 300 and 10 K, a $V_{TH}$ shift as high as +8 V and saturation mobility ($\mu_{sat}$) decrease from 22 to <1 cm^2^/V·s were reported. Godo et al. have shown a $V_{TH}$ shift >4 V between 120 °C and 180 °C also for IGZO TFTs, explained by assuming two kinds of donor-like states as carrier generation sources [11].

Despite the high relevance of these studies to gain insights on device physics, the works in this area are typically based on oxide TFTs annealed at high temperatures (>300 °C), which are more stable than low-temperature ones but are not compatible with low-cost flexible LAE concepts. Furthermore, reports on temperature/stress analysis of circuits based on oxide TFTs or even on other thin-film technologies are scarce. A common-source amplifier static performance with a-Si:H TFTs was reported under bias stress conditions at room temperature, the transfer characteristics of the amplifier being resistant to $V_{TH}$ shift in the TFTs [13]. The current work presents for the first time a unified characterization of low-temperature (180 °C) oxide TFTs and circuits behavior under bias stress and temperatures ranging from 15 to 85 °C. The temperature range was selected having in mind typical storage/utilization environments of general-purpose smart packages and wearables (due to a setup limitation, it was not possible to extend the analysis to lower temperatures; still, as will be seen in the results section, smaller variation of properties are seen as temperature is decreased). From measurements, it was clear that $I_{DS}$ has a strong dependency with temperature and bias stress. However, circuits (inverter and current mirrors) have shown a robust performance by compensating these $I_{DS}$ shifts, even if a low-temperature oxide TFT process is used (180 °C), which leads to lower TFT performance and more device-to-device variation than what is typically obtained with high-temperature processing (>300 °C).

The rest of the paper is structured as follows: Section 2 describes fabrication details of TFTs and circuits. Section 3 presents isolated TFTs stress and temperature-dependent behavior. Section 4 introduces robust circuit topologies that can cancel $I_{DS}$ variations. Section 5 discusses the measured circuit response under bias stress and over a valid range of temperatures, and finally conclusions are drawn in Section 6.

## 2. Transistor and Circuit Fabrication and Characterization

**Figure 1:**
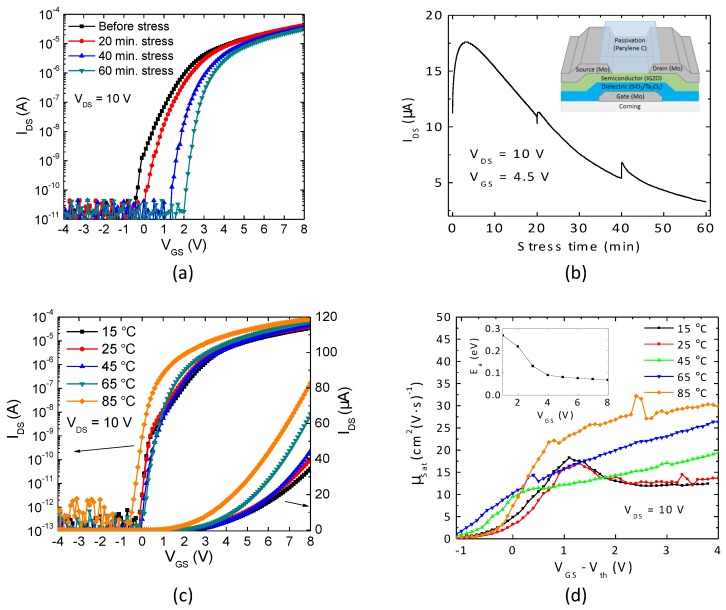
Effect of (**a**,**b**) positive gate and drain bias stress and (**c**,**d**) measurement temperature on the electrical properties of In${}_{2}$O${}_{3}$-Ga${}_{2}$O${}_{3}$-ZnO (IGZO) thin-film transistors (TFTs). (**a**,**c**) show transfer characteristics; (**b**) shows $I_{DS}$ evolution measured continuously during bias stress and the inset presents device structure cross sectional view; (**d**) shows $\mu_{sat}$ dependence of $V_{GS}$-$V_{TH}$ for the different temperatures and the inset presents activation energy with respect to the gate voltage.

In a 2.5 × 2.5 cm^2^ glass substrate, individual TFTs and circuits were fabricated with a staggered bottom gate structure and annealed at 180 °C. Gate, source, and drain electrodes were made of 60 nm-thick Mo deposited in an AJA ATC-1800 sputtering tool (AJA International Inc, North Scituate, MA, USA). The oxide semiconductor was a 30 nm-thick In_2_O_3_-Ga_2_O_3_-ZnO (IGZO) layer, and the dielectric layer was a 175 nm-thick multicomponent/multilayer stack based on Ta_2_O_5_ and SiO_2_. The semiconductor and dielectric layers were deposited by RF magnetron sputtering in an AJA ATC-1300F system (AJA International Inc, North Scituate, MA, USA) without intentional substrate heating. The electrodes and the semiconductor were patterned using a liftoff process, while the dielectric was etched by reactive ion etching in a Trion Phantom 3 system with SF_6_ atmosphere. On top of the devices and after the 180 °C annealing on a hot-plate for 1 h, a 1 μm-thick chemical vapor-deposited parylene-C (poly (monochloro-p-xylylene)) was deposited in a CVD-PDS-2010 tool (Speciality Coating Systems, Indianapolis, IN, USA). The parylene-C layer was deposited on top of an adhesion promoter consisting of Sylane A-147 from Specialty Coating to improve adhesion to IGZO. Access to gate, source, and drain pads was opened using oxygen plasma in the Trion Phantom 3 system (Trion Technology, Inc., Clearwater, FL, USA). This passivation layer improves device stability, as shown in [14,15]. All the TFTs (isolated and integrated in circuits) had a channel length (L) of 20 μm, with channel widths (W) in the range of 40 to 320 μm. A cross-sectional view of the TFT structure is presented in the inset of Figure 1b. Regarding circuits, inverter (common-source amplifier with a diode connected load), 2-TFT, and cascode current mirrors were evaluated.

Electrical characterization of the TFTs and circuits was carried out with an Agilent 4155C semiconductor parameter analyzer (Agilent Technologies, Santa Clara, CA, USA) and a Cascade Microtech M150 probe station with a ERS AC3 chuck for temperature control. All measurements and device stressing were done in the dark.

Regarding bias stress, transistors and circuits were measured before (pre-stressed state) and with a bias stress for every 20 min up to one hour. For isolated TFT, $V_{GS}$ and $V_{DS}$ were set to 4.5 V and 10 V, respectively. To mimic these conditions in inverters, $V_{IN}$ of 4.5 V and $V_{DD}$ of 10 V were used. For two-TFT current mirrors, a constant input current ($I_{IN}$) of 10 μA was supplied, while maintaining the output voltage at 10 V. For the cascode current mirror, output voltage was kept at 15 V instead. For these biasing conditions, the $V_{GS}$ of TFTs in the current mirrors was approximately 4.5 V during stress. Regarding temperature stress, all the devices were allowed to settle at each temperature during 20 min before measurement.

## 3. Isolated TFT Behavior

### 3.1. Stress-Dependent Behavior

Typically, oxide TFTs show positive $V_{TH}$ shift under positive gate bias stress due to charge trapping at the semiconductor/dielectric interface or charge getting into the dielectric [8,9,10]. However, oxide TFTs employing high-k dielectrics can also show an anomalous trend of negative $V_{TH}$ shift, typically attributed to charge detrapping from the dielectric and charge migration by dipole-creation [16,17,18]. Transfer curves measured after discrete periods of gate bias stress are presented in Figure 1a. They suggest a charge trapping mechanism only, with a $V_{TH}$ shift of 1.29 V after 60 min stress. Further insights into the instability mechanism can be seen by analyzing the continuous $I_{DS}$ variation during the entire stress period (Figure 1b). While a decrease of $I_{DS}$ with time is the general trend (in agreement with the positive $V_{TH}$ shift), an opposite behavior is verified during the first minutes of stress. This suggests that in fact the two instability mechanisms mentioned above can be present, but charge trapping tends to dominate for longer periods of stress. The small peaks visible in this plot are due to the short interruptions of bias stress for the measurement of transfer characteristics (20 min and 40 min). A detailed analysis of these competing mechanisms is currently under study.

### 3.2. Temperature-Dependent Behavior

Figure 1c shows the transfer characteristics within a temperature range of 15–85 °C. Three effects are readily observed as temperature increases: (i) $V_{TH}$ is shifted towards negative values; (ii) maximum $I_{DS}$ is increased; (iii) non-idealities appearing at the subthreshold region disappear. The $V_{TH}$ and $I_{DS}$ trends can be explained by the larger concentration of free carriers available, which escape from localized states as temperature is increased [19]. Note that the negative $V_{TH}$ shift is not the only reason for the larger maximum $I_{DS}$; in fact, $\mu_{sat}$ is significantly enhanced as temperature increases (Figure 1d). It is interesting to notice that Chen et al. obtained a considerably smaller increase of field-effect mobility within the same temperature range on IGZO TFTs (from ≈9 to 11 cm^2^/V·s). This can be justified by the different temperatures used for device fabrication: Chen et al. used 300 °C, against 180 °C of our oxide TFTs. It is well known that (post-)processing temperature is one of the most important parameters in setting the quality of IGZO thin films, with lower temperatures resulting in larger density of subgap trap states, hence to more notorious thermal activation of $\mu_{sat}$ [20,21]. This is also related to effect (iii), observed in Figure 1c and also in Figure 1d: As expected, E_a_ decreases with increase in V_GS_ and a minimum of 70 meV is obtained at V_GS_ = 8 V from Figure 1d inset. Values lower than this are typically reported in literature for IGZO TFTs (around 26 meV) [19]. However, it should be noted again that the processing temperature of the present devices is quite low (180 °C) compared to the typical >300 °C reported in literature. As shown in [20], defects close to 100–300 meV are annihilated as annealing temperature increases; hence, it would be expected that devices annealed at lower temperature would present higher E_a_.

Table 1 shows measured $I_{DS}$ with respect to stress and temperature variation, taken for $V_{GS}$ = 4.5 V and $V_{DS}$ = 10 V. Under the considered testing conditions, $I_{DS}$ can be changed by more than 170%, as can be noticed from Table 1.

## 4. Robust Circuits against Bias Stress and Temperature: Theoretical Analysis

**Figure 2:**
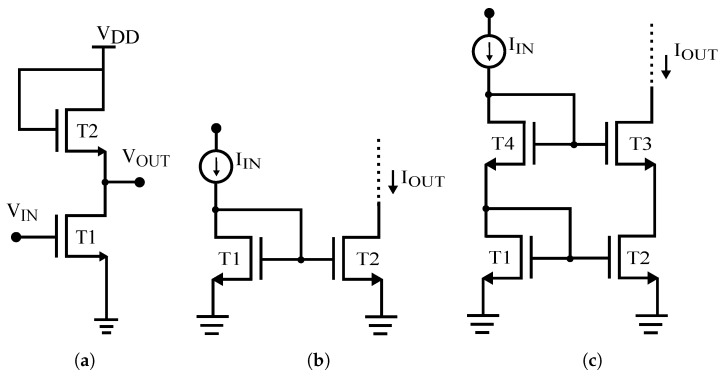
Circuit schematics: (**a**) Inverter; (**b**) Two-TFT current mirror; (**c**) Cascode current mirror.

**Figure 3:**
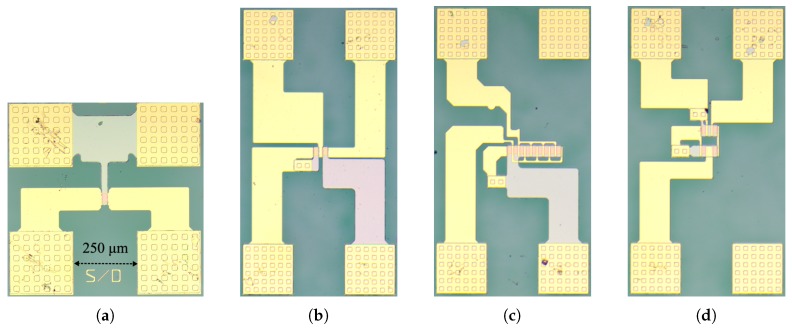
Micrographs of (**a**) TFT; (**b**) Inverter 40–40; (**c**) Two-TFT current mirror with W${}_{T1}$ = 40 μm W${}_{T2}$ = 320 μm; (**d**) Cascode current mirror.

When isolated TFTs show significant performance variation under external stimulus, obviously circuits and systems employing these devices tend to show degradation in their performance. Robust circuit topologies have been analyzed to infer about their ability to counteract $I_{DS}$ variation. Circuit schematics and the corresponding micrographs are presented in Figure 2 and Figure 3, respectively.

For the sake of simplicity, threshold voltage with respect to stress time and temperature variation is expressed as follows:

(1) $$V_{TH}\left(t,T\right)=V_{TH0}+\Delta V_{TH}\left(t,T\right)$$

where *t* is stress time, *T* is temperature, and $\Delta V_{TH}\left(t,T\right)$ is change in the threshold voltage with respect to the stress time and the temperature variation.

**Inverter:** Considering the inverter circuit (Figure 2a), from a large signal analysis perspective, when T1 and T2 are under saturation, and assuming that they have same dimensions and are well matched, (i.e., no significant variation due to process non-uniformity is verified), output voltage ($V_{OUT}$) can be expressed as

(2) $$\begin{array}{c} V_{OUT}=V_{DD}-\frac{\mu_{T1}\left(T\right)}{\mu_{T2}\left(T\right)}V_{IN}-[{\left(\Delta V_{TH}\left(t,T\right)\right)}_{T2}-\frac{\mu_{T1}\left(T\right)}{\mu_{T2}\left(T\right)}\Delta V_{TH}\left(t,T\right){)}_{T1})] \end{array}$$

When T1 and T2 are exposed to same conditions (bias stress and temperature), the output signal is almost independent of the Δ$V_{TH}{\left(t,T\right)}_{T1,T2}$ and mobility variation due to temperature ($\mu_{T1}$≈$\mu_{T2}$).

**Current Mirrors:** When the TFTs are matched, have equal channel length, and ignoring the channel length modulation due to the long L (=20 μm), the input and output currents in this circuit (Figure 2b) are related by

(3) $$\begin{array}{c} \frac{I_{OUT}}{I_{IN}}=\frac{W_{T2}\mu_{T2}\left(T\right){\left(V_{GS}-V_{TH2}\right)}^{2}}{W_{T1}\mu_{T1}\left(T\right){\left(V_{GS}-V_{TH1}\right)}^{2}} \end{array}$$

When all transistors in this circuit are exposed to the same bias stress conditions (it should be noted that $V_{GS}$ of T1 and T2 are equal) and/or temperature variations, $V_{TH}$ variation is going to be the same for both the TFTs, and hence the mirrored current is supposed to be robust against operating conditions. This circuit output resistance is equal to the output resistance of a single TFT ($r_{o}$). Under similar conditions, the same analysis is valid for the cascode current mirror (Figure 2c), and this circuit has high accuracy because of its high output impedance ($g_{m}r_{o}^{2}$). 

## 5. Circuits Measurements and Discussion

**Figure 4:**
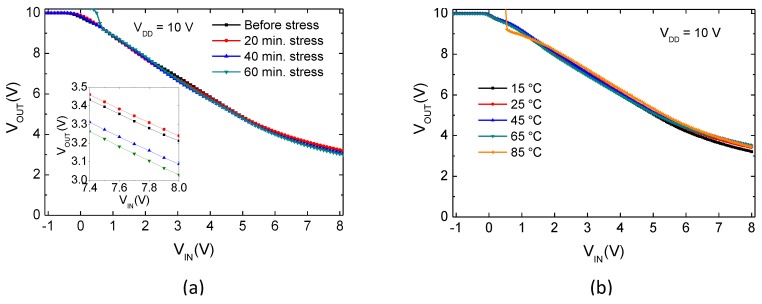
Transfer characteristics of IGZO TFT-based inverters under different (**a**) bias stress (gate + drain) periods and (**b**) temperatures. The inset in (a) shows a magnification at the $V_{OL}$ region.

**Figure 5:**
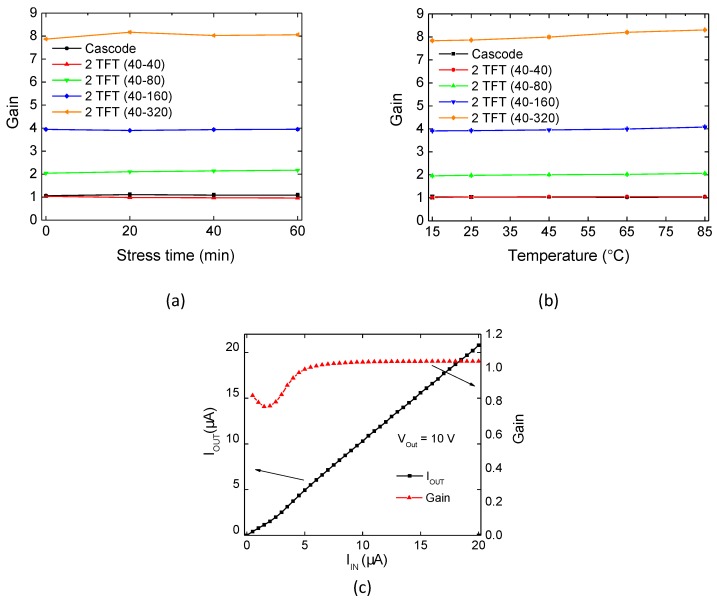
Mirroring ratios of IGZO TFT-based current mirrors under different (**a**) bias stress (gate + drain) periods and (**b**) measurement temperatures. A typical characteristic of a current mirror is presented in (**c**), showing constant gain between $I_{IN}$ = 5–20 μA.

A characterization of inverters and current mirrors under different bias stress periods and temperatures is presented in Figure 4 and Figure 5. The gain of the inverter is approximately given by $\sqrt{\frac{W_{T1}}{W_{T2}}}$, when T1 and T2 have same channel length and good matching. A value of 0.98 was measured, which is close to unity, as expected. As is apparent from Figure 4, when the inverter circuit is subjected to bias stress or temperature, its voltage transfer characteristics are not significantly affected because T1 and T2 are exposed to relatively similar conditions: $V_{TH}$ and mobility variations in one TFT should be canceled by the other, as per (2). The gain of the inverter changed by less than 8% from its original value under bias stress, and less than 5% regarding temperature, as as it can be noticed from Table 2. The inset in Figure 4a shows a magnification of the output voltage when input voltage is high ($V_{OL}$). A decrease of ≈0.2 V on $V_{OL}$ can be noticed between the unstressed state and after 60 min stress. This indicates a decrease of the driver TFT’s (T1) resistance comparatively to the load TFT (T2) as stress time is increased. The effect can be understood by analyzing the bias stress conditions of each transistor: By fixing $V_{IN}$ = $V_{GST1}$ = 4.5 V, $V_{OUT}$ = $V_{DST1}$ = 5.2 V. Hence, since $V_{DD}$ = 10 V, $V_{DST2}$ = $V_{GST2}$ = 4.8 V. With lower $V_{GS}$ and higher $V_{DS}$, the $V_{TH}$ shift for T1 is smaller than for T2 [22,23]. Given that close to $V_{OL}$ T1 is operating in linear regime, R$T_{1}$/R$T_{2}$ is decreased for longer stress periods. The small increase of $V_{OL}$ between the unstressed state and after 20 min stress might be explained by considering that the initial period where the anomalous $V_{TH}$ shift is predominant (Figure 1b) is found to have some variation from device to device. Hence, until the 20 min stress period, the anomalous $V_{TH}$ shift (negative) in T2 might be slightly more significant than in T1.

For the current mirrors, gain with respect to bias stress and temperature is presented in Figure 5. This gain as well as its variation relative to unstressed states and room temperature measurements are reported in Table 3. Current mirrors with different $\frac{W2}{W1}$ are presenting close-to-theoretical gain, without significant changes under bias stress or temperature, as expected per (3). A maximum variation below 9% was measured with increasing stress time, while for temperature variation it was always below 5%. Proving its robustness, the cascode current mirror presents higher stability than the two-TFT configurations due to its high output impedance. The presented gains are taken as an average of gain between $I_{IN}$ = 5–20 μA, with the standard deviation over this range never exceeding 2%. Figure 5c illustrates this by showing for the two-TFT (40–40) current mirror that $I_{OUT}$ is increasing linearly with $I_{IN}$, resulting in a constant gain over this range. This is verified for all the current mirrors, regardless of bias stress and temperature. It should be noted that the circuits have shown a robust performance, irrespective of the unavoidable mismatches arising due to the low-temperature fabrication and different layouts of the TFTs [24] (i.e., direct and fingered layouts, see Figure 3).

## 6. Conclusions

This paper presented an application-oriented analysis of oxide TFTs under gate and drain bias stress and different measurement temperatures. The relatively large changes of $I_{DS}$ (as high as 172%) under the more severe conditions (i.e., longer bias stress period and higher temperature) are significantly attenuated on circuits, namely inverters (or common-source amplifier with a diode-connected load) and current mirrors, given that the variations seen in one node are counteracted by the ones in other node. Thus, this work shows that even when employing low-temperature oxide TFT processes (which inherently result in higher device-to-device variation and degraded TFT stability compared to higher temperature processes), oxide TFT circuitry following simple design considerations can provide robust performance in real-world applications demanding continuous operation.

## Figures and Tables

**Table 1:** Drain current variation on IGZO TFTs under different durations of gate and drain bias stress and measurement temperature. $I_{DS}$ measured at $V_{GS}$ = 4.5 V and $V_{DS}$ = 10 V.

	Stress (Min.)				Temperature (°C)				
	**0**	**20**	**40**	**60**	**15**	**25**	**45**	**65**	**85**
$I_{DS}$ (μA)	11.3	10.7	6.8	5.0	6.8	8.1	7.1	11.5	21.9
Relative variation (%)	0.0	−5.2	−39.5	−56.0	−15.5	0.0	−12.2	43.3	172.3

**Table 2:** Inverter maximum gain variation with respect to bias stress and temperature variation.

	Stress (Min.)				Temperature (°C)				
	**0**	**20**	**40**	**60**	**15**	**25**	**45**	**65**	**85**
Gain	0.99	0.95	0.94	0.92	0.98	0.98	0.97	0.94	0.97
Relative variation (%)	0.0	−4.0	−4.9	−7.3	0.1	0.0	−1.2	−4.3	−1.3

**Table 3:** Current mirror average gain variation under different durations of gate + drain bias stress and measurement temperature, when $I_{in}$ is varied from 5 to 20 μA.

Gain	Stress (Min.)				Temperature (°C)				
	0	20	40	60	15	25	65	45	85
Cascode	1.06	1.11	1.09	1.09	1.04	1.04	1.04	1.02	1.04
Relative variation (%)	0.0	4.7	2.8	2.8	−0.2	0.0	0.0	−1.7	0.0
40–40	1.03	0.96	0.95	0.94	1.02	1.04	1.05	1.05	1.05
Relative variation (%)	0.0	−6.6	−7.9	−8.7	−1.9	0.0	1.0	1.0	1.0
40–80	2.03	2.12	2.15	2.19	1.96	1.98	2.01	2.02	2.07
Relative variation (%)	0.0	4.1	6.0	7.6	−1.1	0.0	1.3	2.1	4.2
40–160	3.95	3.85	3.86	3.86	3.91	3.92	3.95	3.99	4.08
Relative variation (%)	0.0	−2.5	−2.4	−2.4	−0.2	0.0	0.8	1.8	4.0
40–320	7.90	8.15	7.85	7.88	7.84	7.86	7.99	8.20	8.30
Relative variation (%)	0.0	3.2	−0.6	−0.2	−0.4	0.0	1.7	4.3	5.6

## Abbreviations

The following abbreviations are used in this manuscript:

a-IGZO	amorphous Indium Gallium Zinc Oxide
TFT	Thin-film Transistor
NFC	Near-field communication
a-Si:H	Hydrogenated amorphous silicon
RF	Radio Frequency
RT	Room Temperature
LAE	Large area electronics
